# Characterization of bacteriophages infecting multidrug-resistant uropathogenic *Escherichia coli* strains

**DOI:** 10.1007/s00705-024-06063-x

**Published:** 2024-06-08

**Authors:** Barbora Markusková, Sulafa Elnwrani, Michal Andrezál, Tatiana Sedláčková, Tomáš Szemes, Lívia Slobodníková, Michal Kajsik, Hana Drahovská

**Affiliations:** 1 University Science Park, Comenius University in Bratislava, Bratislava, Slovakia; 2 Department of Molecular Biology, Faculty of Natural Sciences, Comenius University in Bratislava, Bratislava, Slovakia; 3 Institute of Microbiology, Faculty of Medicine, Comenius University in Bratislava and University Hospital Bratislava, Bratislava, Slovakia

**Keywords:** bacteriophages, phage therapy, UTI, *Escherichia coli*, UPEC

## Abstract

Uropathogenic *Escherichia coli* (UPEC) is the most common causative agent of urinary tract infections, and strains that are resistant to antibiotics are a major problem in treating these infections. Phage therapy is a promising alternative approach that can be used to treat infections caused by polyresistant bacterial strains. In the present study, 16 bacteriophages isolated from sewage and surface water were investigated. Phage host specificity was tested on a collection of 77 UPEC strains. The phages infected 2–44 strains, and 80% of the strains were infected by at least one phage. The susceptible *E. coli* strains belonged predominantly to the B2 phylogenetic group, including strains of two clones, CC131 and CC73, that have a worldwide distribution. All of the phages belonged to class *Caudoviricetes* and were identified as members of the families *Straboviridae*, *Autographiviridae*, and *Drexlerviridae* and the genera *Kagunavirus*, *Justusliebigvirus*, and *Murrayvirus*. A phage cocktail composed of six phages – four members of the family *Straboviridae* and two members of the family *Autographiviridae* – was prepared, and its antibacterial activity was tested in liquid medium. Complete suppression of bacterial growth was observed after 5–22 hours of cultivation, followed by partial regrowth. At 24 hours postinfection, the cocktail suppressed bacterial growth to 43–92% of control values. Similar results were obtained when testing the activity of the phage cocktail in LB and in artificial urine medium. The results indicate that our phage cocktail has potential to inhibit bacterial growth during infection, and they will therefore be preserved in the national phage bank, serving as valuable resources for therapeutic applications.

## Introduction

*Escherichia coli*, a member of the family *Enterobacteriaceae*, is the most common causative agent of urinary tract infections (UTIs) worldwide [1, 2]. Despite commensal colonization of the lower intestine of warm-blooded animals, its presence in the urinary tract can lead to acute UTIs and even to recurrent or chronic infections [3]. The uropathogenic *E. coli* (UPEC) are a broad group of strains with heterologous genomes that carry a diverse set of virulence genes that help these strains to invade the urothelium [1, 4]. The majority of UPEC isolates belong to phylogenetic group B2 and, to a lesser extent, to the D, A, and B1 phylogroups [5–7]. In particular, one lineage of phylogroup B2 with the sequence type (ST) ST131 is emerging and becoming widespread globally [8, 9]. The success of ST131 possibly may lie in the multidrug resistance of many of its representatives [10]. An expansion of antibiotic resistance has been also reported among other UPEC strains [3, 11]. Therefore, the need for alternatives to antibiotics for treatment of UTIs is increasing. Phage therapy is one of the strategies that can be used to treat infections caused by polyresistant bacterial strains [12]. Lytic bacteriophages attack bacteria in a strain-specific manner, independently of their antibiotic resistance, and they do not have any of the side effects that are usually associated with antibiotic treatment, e.g., disturbance of the natural microbiota [13]. Phage therapy may also be useful for patients with chronic or recurrent urinary tract infections, which are most common in females. Although these infections are frequently caused by bacterial agents that are susceptible to antibiotics, phage therapy may spare the patients repeated courses of antimicrobial therapy and their inevitable side effects. During therapy, bacteriophages can be used alone or combined with antibiotics, which are usually applied systemically, while the phages are applied locally, directly to the infectious focus [14].

A major advantage of phage therapy is the ease with which therapeutic phages can be isolated from the environment. However, it is crucial to select phages that have a suitable host range, capable of effectively targeting and killing a specific pathogen of interest. Only strictly lytic phages that are incapable of lysogeny should be used for phage therapy [15]. Well-characterized phages that meet these criteria are often deposited in phage banks, serving as valuable resources for therapeutic or industrial applications [16].

Recently, we isolated and characterized two bacteriophages with broad host specificity against a panel of local uropathogenic *E. coli* strains [17]. In the present study, we contributed further to the establishment of a national phage bank by characterizing additional phages covering a broader spectrum of *E. coli* strains. Six phages with desirable properties were combined into a phage cocktail, and its antibacterial activity was measured in liquid artificial urine medium.

## Materials and methods

### Bacterial strains and culture conditions

In total, 76 *E. coli* isolates obtained from the urine of ambulatory or hospitalized patients with symptomatic bacteriuria [17] and reference *E. coli* strain CFT073 [18] were used in the study. Strains were classified into phylogenetic groups by quadruplex PCR [19] and into CH types by two-locus Sanger sequencing [20], and some strains were also analyzed by whole-genome sequencing [21]. Bacteria were cultivated overnight at 37°C on stationary Luria-Bertani (LB) plates or in LB broth with shaking at 220 rpm.

### Bacteriophage isolation and propagation

Novel bacteriophages were isolated from wastewater or surface water by procedures that have been described previously [17]. Briefly, 10 ml of water was sterilized by passage through a 0.22-µm filter and mixed with an equal volume of twofold-concentrated LB medium supplemented with 0.5% glucose, 2.5 mM CaCl_2_, and 2.5 mM MgCl_2_ and 200 µL of overnight bacterial culture. The inoculated mixture was cultivated overnight at 37°C with shaking. The phage lysate was sterilized by adding 100 µL chloroform, and bacteria were removed by centrifugation at 9000 rpm for 30 min. Phages were purified through three repeated isolations from single plaques on double agar and then enriched by infecting an exponentially growing indicator strain at a multiplicity of infection (MOI) of 0.001, followed by overnight cultivation. The medium was removed, and phage particles were concentrated by polyethylene glycol (PEG) precipitation (10% PEG 600; 1 M NaCl) and resuspended in SM buffer (100 mM NaCl, 8 mM MgSO_4_, 50 mM Tris-HCl, pH 7.5, and 0.002% gelatin) for long-term storage at 4°C. Prior to DNA isolation, phages were purified by cesium chloride gradient centrifugation, using 1.1, 1.3, and 1.5 mg/ml CsCl solutions. After 3 hours of centrifugation at 22,000 rpm at 10°C, a light blue phage band was transferred to dialysis tubing with a molecular weight cutoff of 10 kDa and dialyzed against SM buffer to remove excess salt. Two previously described phages (vKMB22 and vKMB26) [17] were also included in the present study.

### Phage and bacterial genome sequencing and analysis

Phage and bacterial DNA were isolated using a Norgen Phage DNA Isolation Kit and a Canvas DNA Isolation Kit, respectively. Bacterial and phage genome libraries were prepared using a Nextera XT Library Preparation Kit according to a standard protocol. Paired-end 2×300-bp libraries were sequenced on an Illumina MiSeq platform. Reads were deduplicated and assembled *de novo* using the SPAdes 3.10.1 genome assembler. Annotation of genomes was done using the RASTtk server [22]. The functions of hypothetical proteins were predicted using HHpred [23]. Bacteriophages were classified using the Patric Taxonomy Classification Service [24], which uses k-mer matches, and were subsequently subjected to genome zeroing based on a reference phage of a given genus (ICTV taxonomy). Visualization and comparison of phage genomes was done in EasyFig 2.2.5 and in Geneious 11.1.5 (https://www.geneious.com). Nucleotide similarity (NS) of genomes was calculated by using VIRIDIC [25]. The web-based programs VirulenceFinder [26] and ResFinder [27] were used to find bacterial resistance and virulence genes in phage genomes. Lysogenic traits such as repressor, recombinase, and attachment sites were browsed manually in phage annotations and with the aid of the web-based program Phaster [28]. Predicted ORFs with known protein homologs were divided into five groups: structural proteins, enzymes and proteins for DNA replication/modification/regulation, packaging proteins, host lysis proteins, and additional proteins. The sequences of the phages were deposited in the GenBank database under the accession numbers shown in Table 1.

**Table 1:** List of isolated phages

Name	Short name	Family	Genus	Reference phage (% similarity)	Genome length (kbp)	No. of CDSs	No. of tRNAs	Accession number
vB-EcoP_KMB47	vKMB47	*Autographiviridae*	*Vectrevirus*	VEc3 (89)	44.5	54	-	OR424385
vB-EcoP_KMB14	vKMB14	*Autographiviridae*	*Vectrevirus*	VEc3 (90)	44.6	56	-	OR424384
vB-EcoS_KMB25	vKMB25	*Drexlerviridae*	*Tlsvirus*	Stevie (93)	50.1	101	-	OR424386
vB-EcoS_KMB46	vKMB46	*Drexlerviridae*	*Warwickvirus*	swan01 (94)	51	98	-	OR525699
vB-EcoM_KMB37	vKMB37	*-*	*Justusliebigvirus*	phi92 (92)	148.6	296	12	OR525700
vB-EcoM_KMB43	vKMB43	*Straboviridae*	*Krischvirus*	RB49 (92)	164.5	292	-	OR525698
vB-EcoM_KMB38	vKMB38	*Straboviridae*	*Mosigvirus*	RB69 (94)	169.6	283	2	OR525701
vB-EcoM_KMB22	vKMB22	*Straboviridae*	*Tequatrovirus*	T4 (85)	167	288	10	OR539217
vB-EcoM_KMB23	vKMB23	*Straboviridae*	*Tequatrovirus*	T4 (86)	151.5	254	9	OR525702
vB-EcoM_KMB26	vKMB26	*Straboviridae*	*Tequatrovirus*	T4 (85)	168.2	291	9	OR539218
vB-EcoM_KMB39	vKMB39	*Straboviridae*	*Tequatrovirus*	T4 (84)	168.9	280	9	OR539219
vB-EcoM_KMB40	vKMB40	*Straboviridae*	*Tequatrovirus*	T4 (84)	167	286	11	OR539220
vB-EcoM_KMB42	vKMB42	*Straboviridae*	*Tequatrovirus*	T4 (83)	166	280	10	OR539221
vB-EcoP_KMB41	vKMB41	*-*	*Murrayvirus*	EC2 (78)	41.8	64	-	OR539216
vB-EcoS_KMB35	vKMB35	*-*	*Kagunavirus*	K1G (64)	43.9	83	-	OR525704
vB-EcoS_KMB36	vKMB36	*-*	*Kagunavirus*	K1G (63)	44.7	86	-	OR525703

### Determination of phage host range, growth curve, and phage adsorption

The susceptibility of bacterial isolates to the phages and the efficiency of plating (EOP) were tested by dropping 10 µl of phage dilutions in SM buffer onto double-layer agar plates. A strain was considered susceptible if single plaques were formed. The relative EOP of a phage was defined as strong, moderate, and weak when single plaques were observed at a concentration of 10^4^, 10^6^, and 10^8^ plaque-forming units (PFU)/ml, respectively. An infection was considered abortive if a lysis zone appeared without plaque formation. The phage host range test was repeated twice.

A one-step growth curve was generated by adding 100 µl of 10^7^ PFU/ml phage lysate to 10 ml of exponentially growing bacterial culture. Phages were allowed to adsorb for 10 min at 37°C, after which the mixture was then centrifuged and the pellet was resuspended in 10 ml of prewarmed LB. At this time, 1-ml samples were taken every 5 min, and the phages were counted by plaque assay.

Phage adsorption was measured by adding 20 µl of 10^8^ PFU/ml phage suspension to 180 µl of overnight bacterial culture and allowing the phage to adsorb for 5 min at 37°C. Subsequently, 10 µl of sample was diluted in 1 ml of SM buffer (100 mM NaCl, 8 mM MgSO_4_, 50 mM Tris-HCl, pH 7.5, and 0.002% gelatin), adsorbed phages were removed by centrifugation, and unadsorbed phages were counted by plaque assay. The measurements were repeated in triplicate.

### Effect of phage cocktail on bacterial culture in liquid medium

Artificial urine (pH 6.5) was prepared as described previously [29] with some modifications. In order to avoid precipitation, the concentration of ureic acid was reduced to 0.4 mM, and to support bacterial growth, 0.1% peptone and 0.01% yeast extract were included [30]. An overnight bacterial culture was washed with PBS, diluted in LB medium or artificial urine to an OD_580_ of 0.1 (approx. 8 × 10^6^ colony-forming units (CFU)/ml). Phage cocktails composed of six phages (Table 2) with concentrations of 3 × 10^9^ PFU/ml (MOI = 100), 3 × 10^8^ PFU/ml (MOI = 10), and 3 × 10^7^ PFU/ml (MOI = 1) of each phage were prepared in PBS. An aliquot of 190 µl of bacterial culture and 10 µl of the phage cocktail were added to each well of a 96-well microtiter plate, and the inhibitory effect of the cocktail was evaluated by monitoring the optical density (OD_580_) in a Varioskan reader (Thermo Fisher) during 24 h of incubation at 37°C with shaking at 220 rpm. Each sample was tested in triplicate in three independent experiments (n = 9). The percentage of bacterial growth inhibition was calculated as the total area under the curve of the positive control minus the total area under the curve of the sample with phage cocktail, divided by the total area under the curve of the positive control × 100 [31]. The results are reported as the average relative bacterial growth inhibition from three experiments.

**Table 2:** Growth parameters of phages selected for the phage cocktail

Phage	Genus	Propagating strain (PhyG, ST)	Adsorption (%)	Latent period (min)	Burst size (PFU/cell)	Sensitivity to strains used for cocktail evaluation^1^						
						CFT 073	KMB -718	KMB -735	KMB -706	KMB -507	KMB -709	KMB -704
vKMB14	*Vectrevirus*	KMB-528 (B2, ST131)	98	7,5	190	0	4	4	0	0	4	0
vKMB47	*Vectrevirus*	CFT073 (B2, ST73)	98	10	211	4	4	4	0	0	4	0
vKMB43	*Krischvirus*	CFT073 (B2, ST73)	80	10	38	4	4	4	4	4	4	1
vKMB23	*Tequatrovirus*	KMB-507 (B2, ST420)	72	10	95	0	4	4	4	4	0	0
vKMB26	*Tequatrovirus*	KMB-517 (B2, ST131)	81	15	47	4	4	3	4	4	4	0
vKMB42	*Tequatrovirus*	KMB-233 (B1, ST9013)	77	15	69	0	3	4	4	3	3	0

^1^Phage sensitivity: strong (4), moderate (3), weak (2), lysis without plaques (1), no infection (0)

## Results

### Phage isolation and host specificity

**Fig. 1:**
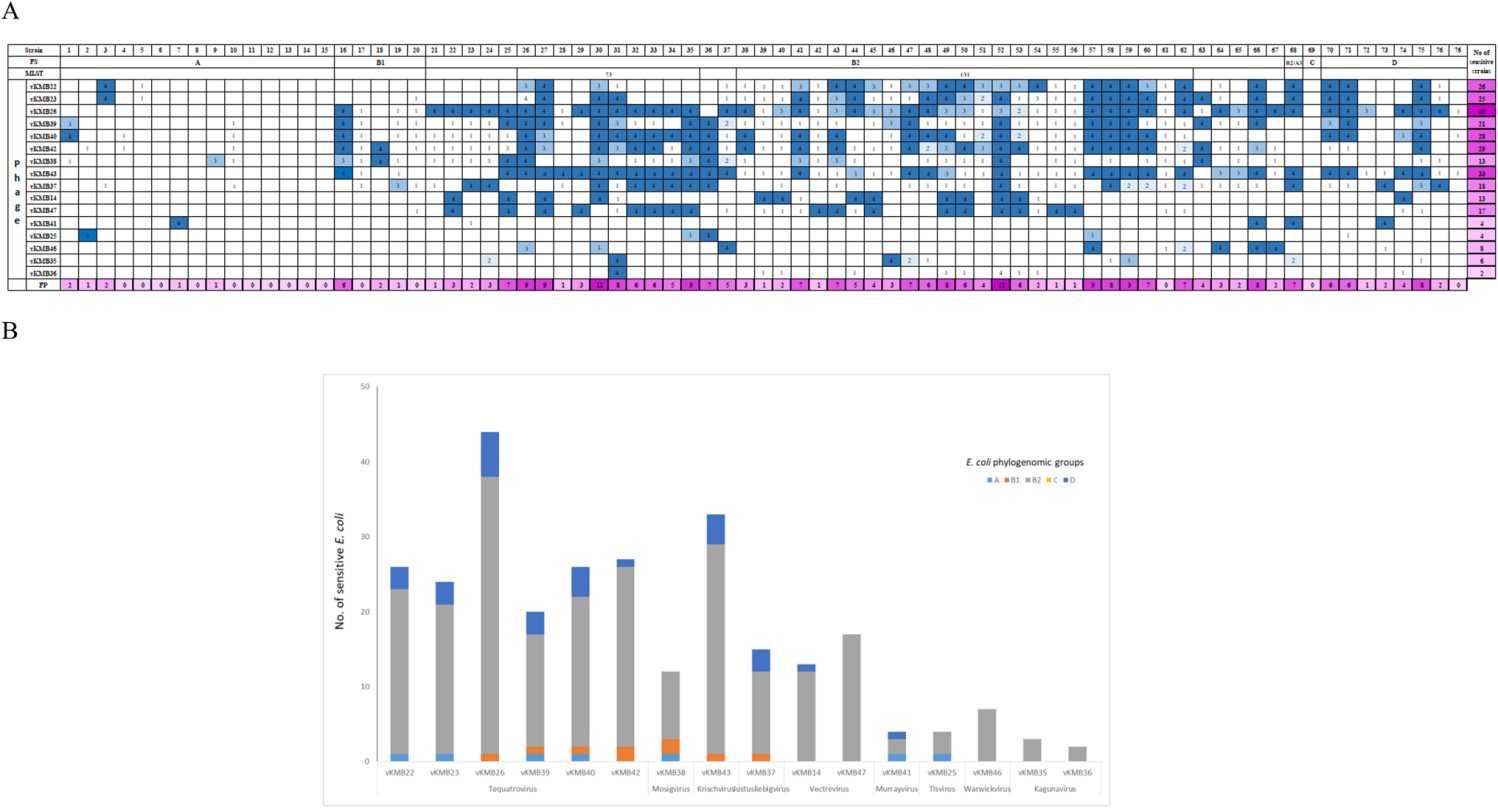
Host specificity of phages against a panel of UPEC strains. (A) Sensitivity of 77 UPEC strains to the isolated phages. Legend: strong (4), moderate (3), weak (2), lysis from without (1). (B) Host spectrum of isolated phages according to phylogenetic groups; strains with EOP > 0.01% were counted

In the present study, sixteen different bacteriophages infecting UPEC were isolated from sewage or surface water and characterized (Table 1). The phage host specificity was tested on a collection of 77 clinical *E. coli* strains belonging to different phylogenetic lineages and sequence types. The phages were able to infect 2–44 (3–57%) of the strains (Fig. 1). The broadest host specificity was observed for the phages vKMB26 and vKMB43, which lysed 44 and 33 (57 and 43%) of the strains, respectively. Sixty-two strains (80%) were infected by at least one phage, and *E. coli* KMB-735, the most sensitive strain, was infected by 12 different phages (Fig. 1). Susceptible *E. coli* strains belonged predominantly to the B2 phylogenetic group, including strains of clones CC131 and CC73, which have spread worldwide, and all but one strain of this group were susceptible to at least one phage (Fig. 1A). High sensitivity was also observed in strains belonging to phylogenetic group D (89% of the susceptible strains). Limited phage sensitivity was observed with strains of the A and B1 phylogroups, with 35% on average being vulnerable to at least one phage. One strain belonging to phylogenetic group C was resistant to all of the phages.

### Genome sequencing of bacteriophages

Phage genome sequences were assembled *de novo* with a high level of contig coverage (> 300). All of the phages possessed a dsDNA genome and belonged to the class *Caudoviricetes*. The majority of them were classified as members of the family *Straboviridae* (T4-like phages) (n = 8), and the others belonged to the families *Autographiviridae* (n = 2) and *Drexlerviridae* (n = 2) and the genera *Kagunavirus* (n = 2), *Justusliebigvirus* (n = 1), and *Murrayvirus* (n = 1).

Genome sequence alignment to the reference phages of a given genus showed a high level of sequence similarity. However, based on the > 95% sequence identity cutoff for species demarcation by the ICTV [32], most of these phage isolates should be classified as members of new species (Table 1). Phages vKMB26 (genus *Tequatrovirus*) and vKMB47 (genus *Vectrevirus*) were the exceptions, showing 97% and 98% sequence identity to phages vB_EcoM-G3G7 (MZ234040.1) and vB_EcoP-101101UKE1 (MZ234012.1), respectively. The highest variability in the phage genomes was displayed in genes associated with receptor recognition. Except for phage vKMB25 (genus *Tlsvirus*), all of the phages were strictly lytic, and none of them encoded bacterial resistance or virulence genes.

### Family *Straboviridae*

**Fig. 2:**
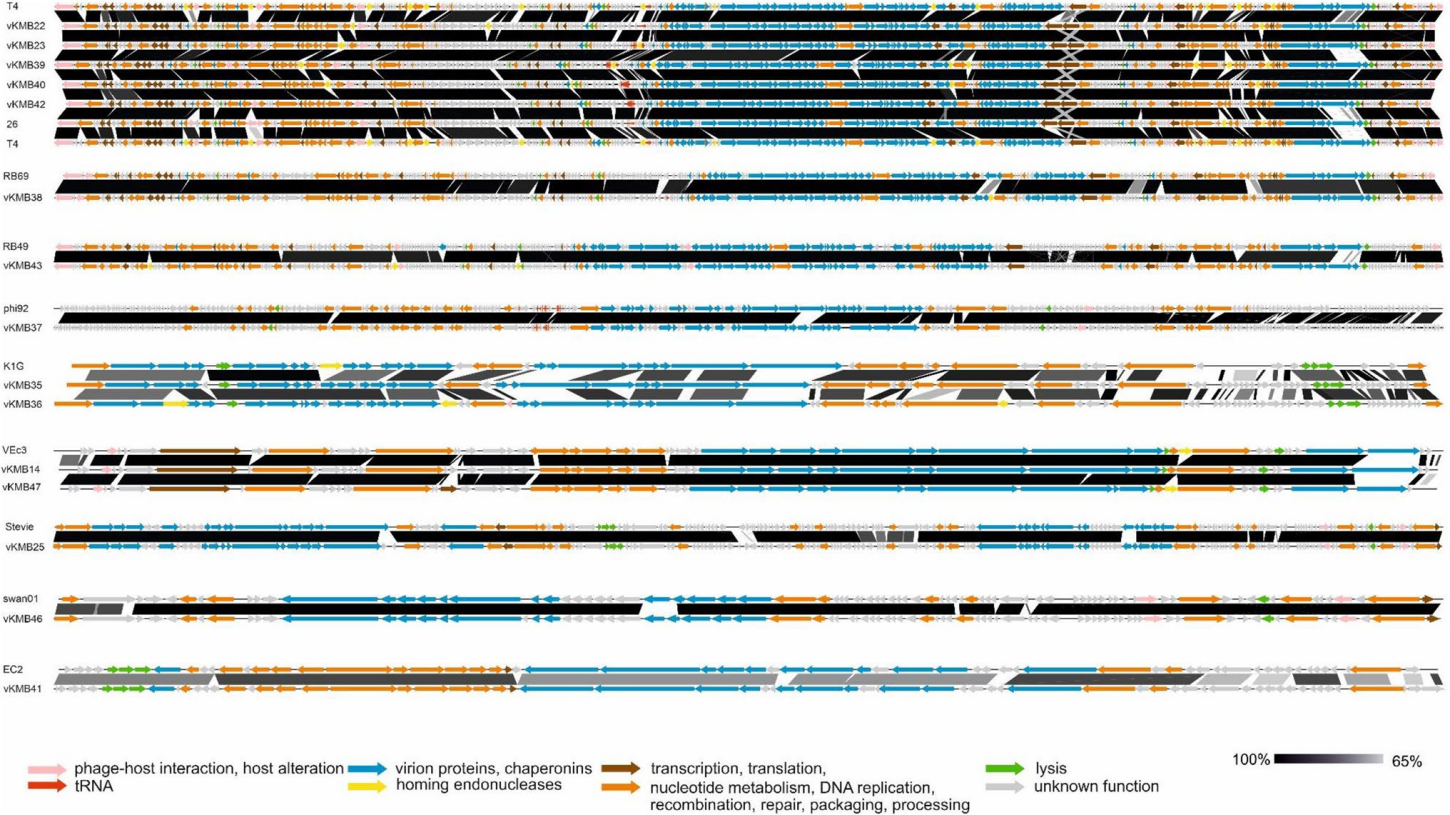
Genome comparison of the new phages with the reference genomes. Comparisons were made in EasyFig

The eight novel *Straboviridae* phages in the collection exhibited the most extensive host range. Individually, these phages infected 17–56% of the UPEC strains, and collectively, they infected 73% of them. These novel phages were classified into three genera. Six phages were categorized as members of the genus *Tequatrovirus*, sharing a genome organization similar to those of T-even phages, with 83–86% nucleotide sequence identity to *Escherichia* phage T4 (NC_000866.4) (Fig. 2). While these six phages exhibited similarities in their genetic makeup, notable differences were observed in the sequences of the gp36, gp37, and gp38 long tail fiber proteins, the gp12 short tail fiber protein, and the RNA polymerase and the ADP ribosyltransferase, and they differed in the presence of homing endonuclease genes and genes with unknown functions. The *Tequatrovirus* phages formed three groups based on the organization of their long tail fiber genes and the similarity of their host range. The first group comprised vKMB22 and vKMB23, while the second group consisted of three phages: vKMB39, vKMB40, and vKMB42. vKMB26, which displayed the broadest host range within the entire collection (58%), was the most distantly related.

Another member of the family *Straboviridae*, phage vKMB38, was classified as a member of the genus *Mosigvirus* and exhibited 94% sequence identity to phage RB69 (NC_004928.1) (Fig. 2). Significant differences between RB69 were observed in the dihydrofolate reductase gene *frd* (34% identity), the capsid decoration protein gene *hoc* (29% identity), and the receptor binding protein gp37 gene (70% identity). Despite possessing promising characteristics typically associated with highly virulent phages, vKMB38 demonstrated a narrower host range, being able to infect only 17% of the tested UPEC strains. Of the *Straboviridae* family members tested, it had the most limited host range.

Phage vKMB43 displayed a close relationship to the pseudo-T-even krischvirus RB49 (NC_005066.1), with 92% nucleotide sequence identity (Fig. 2). Unlike the other members of the family *Straboviridae*, vKMB43 lacks any tRNA genes, which is a characteristic feature of members of the genus *Krischvirus*. Notably, vKMB43 demonstrated a wide host range, infecting 43% of the tested strains, predominantly within the B1, B2, and D phylogroups.

### Genus *Justusliebigvirus*

Phage vKMB37 has a 148-kbp-long genome and is related to members of the genus *Justusliebigvirus*, namely phi92 (NC_023693.1) (Fig. 2), *Escherichia* phage inny (MN850601.1), and VEcB (NC_052663.1). These phages represent a distinct branch within the order *Caudoviricetes* and are placed within the subfamily *Stephanstirmvirinae*. Like other *Justusliebigvirus* phages, vKMB37 contains numerous tRNA genes (12) and several tail spike proteins. However, vKMB37 differs from phi92 in that the K1-specific neuraminidase is replaced by another putative glycosidase in vKMB37. This phage was able to infect 23% of the tested strains, primarily within the B1, B2, and D phylogroups.

### Genus *Kagunavirus*

Two phages, vKMB35 and vKMB36, were identified as members of the genus *Kagunavirus* within the subfamily *Guernseyvirinae*. These phages have collinear genomes approximately 44 kbp in length, displaying low nucleotide sequence similarity to each other as well as to the reference phage K1G (NC_027993.1) (64% identity). Notably, the genomes of these phages exhibited a high degree of mosaicism, with regions of notable similarity being interrupted by non-similar homologous genes (Fig. 2). Both vKMB35 and vKMB36 displayed a narrow host range, with vKMB35 infecting six (8%) strains, while vKMB36 infected only two (3%) strains belonging to the B2 phylogroup.

### Genus *Vectrevirus*

Two phages, vKMB14 and vKMB47, were identified as members of the genus *Vectrevirus* within the family *Autographiviridae*. These phages possess 44-kbp genomes exhibiting a typical T7-like organization. Their genome sequences are 89% identical to each other and to the reference phage VEc3 (NC_047899.1) (Fig. 2). The genomes of vKMB14 and vKMB47 differ primarily in the tail spike region. Notably, both phages form clear plaques with halo zones, suggesting the presence of a polysaccharide depolymerase in the phage receptor binding protein. In terms of host range, vKMB14 and vKMB47 were able to infect 17% and 22% of the tested strains, respectively. The host spectrum partially overlapped, with the sensitive strains predominantly belonging to the B2 phylogroup.

### Family *Drexlerviridae*

Two of the phages belonged to the family *Drexlerviridae*. vKMB25, with a 50-kbp genome, was found to be highly similar to the tlsvirus Stevie (NC_027350.1, 90.5% identity, Fig. 2). We found some features of vKMB25 that could be associated with temperate phages, one of them being a potential prophage insertion site (attL/attR) with the nucleotide sequence GTTGACGGCGTG. vKMB25 was also found to encode a homolog of the superinfection exclusion Cor lipoprotein, which protects a lysogenized host from superinfection by inactivating the phage receptor. Despite the fact that no repressor or integrase genes were detected, there is a chance that vKMB25 could be a temperate phage and thus unsuitable for therapeutic applications. Moreover, its host range was found to be low, infecting only 5% of the tested strains.

Another member of this family, vKMB46, was highly similar to swan01 phage (genus *Warwickvirus*) (NC_048202.1), with 92% nucleotide sequence identity and a similar genome length of 50 kbp (Fig. 2). Most of the predicted proteins, especially in the 18 kbp-region upstream of the terminase gene, had no assigned function, even after re-annotation using HHpred. The vKMB46 structural genes are organized similarly to those of the temperate phage lambda (NC_001416.1), although the genes required for a lysogenic cycle were not present. Phage vKMB46 infected 10% of the strains tested.

### Genus *Murrayvirus*

Phage vKMB41 was identified as a member of the genus *Murrayvirus*. The reference phage EC2 (genus *Murrayvirus*; NC_047742.1) shared 73% sequence identity with vKMB41 (Fig. 2). Phages of this genus typically have a relatively small genome (approx. 42 kbp) and high GC content (> 59%). vKMB41 was found to have narrow host specificity, infecting 5% of the tested strains.

### Inhibition of *E. coli* growth by a phage cocktail

Based on their host range, genome features, and growth properties, a phage cocktail composed of six phages – four *Straboviridae* members and two *Autographiviridae* members – was prepared (Table 2). Altogether, these phages were able to productively infect 52 (67%) of the tested strains. The selected phages had a high adsorption rate (72–98% in 5 min), a short latent period (7.5–15 min), and relatively high burst size (38–211) when measured on the propagating strain (Table 2). The efficiency of the cocktail was tested on a panel of seven *E. coli* strains belonging to frequently detected UPEC clones. Double agar plate testing showed that the phages were highly infectious for six strains, as these strains were productively infected by 3–6 phages from the cocktail. In addition, *E. coli* KMB-704 (ST69), a strain that is resistant to almost all phages, was also included in the assay.

**Fig. 3:**
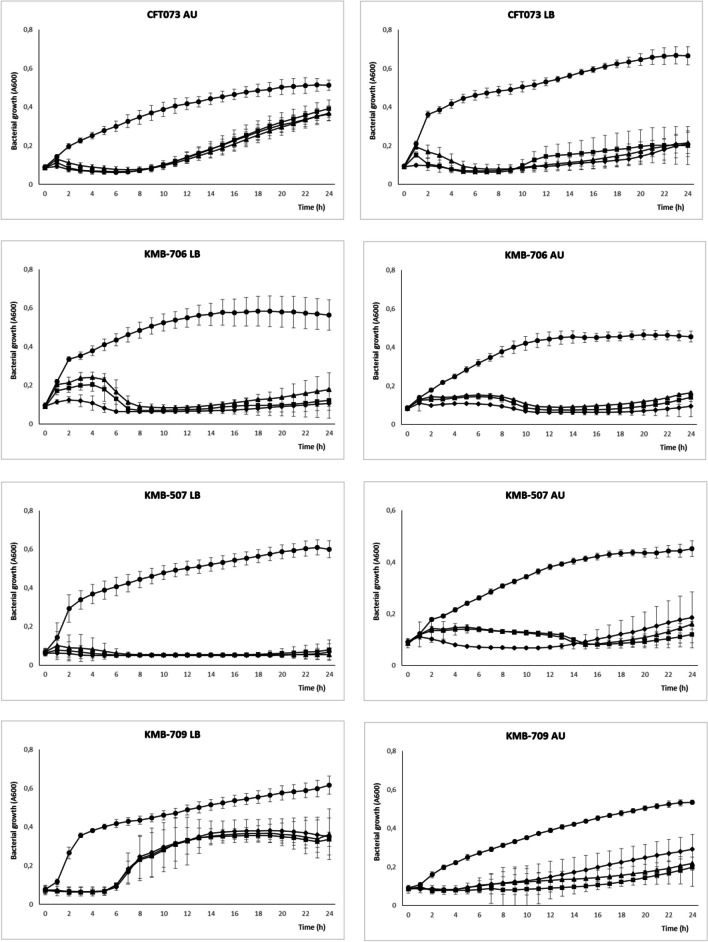

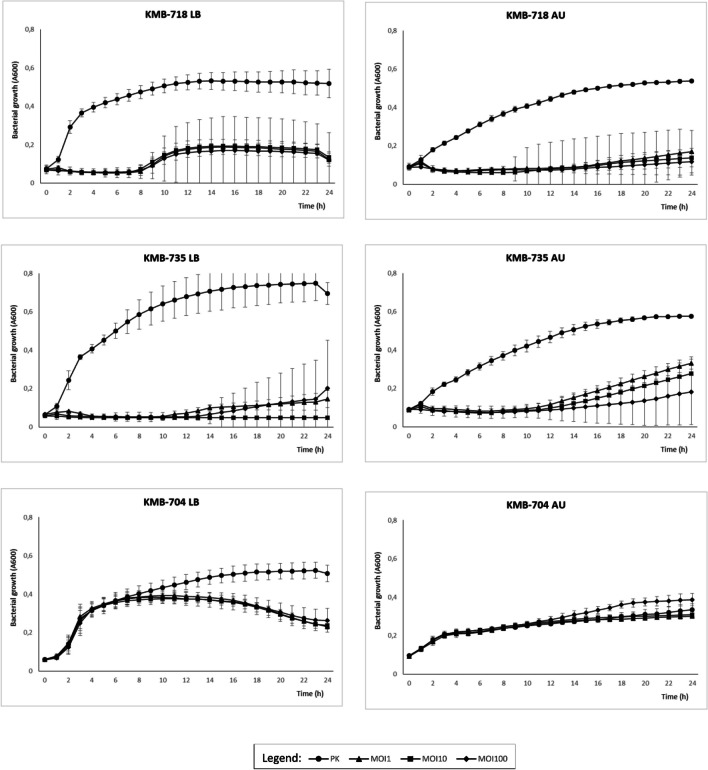
Bacterial growth curves in the presence of phage cocktail in LB and AU media. Growth inhibition was observed as a decrease in optical density compared to control without phages. Three different MOIs were used: 1 (triangle), 10 (square), 100 (diamond), and none in control (circle). Results are presented as the mean of three measurements replicated in three independent experiments

**Fig. 4:**
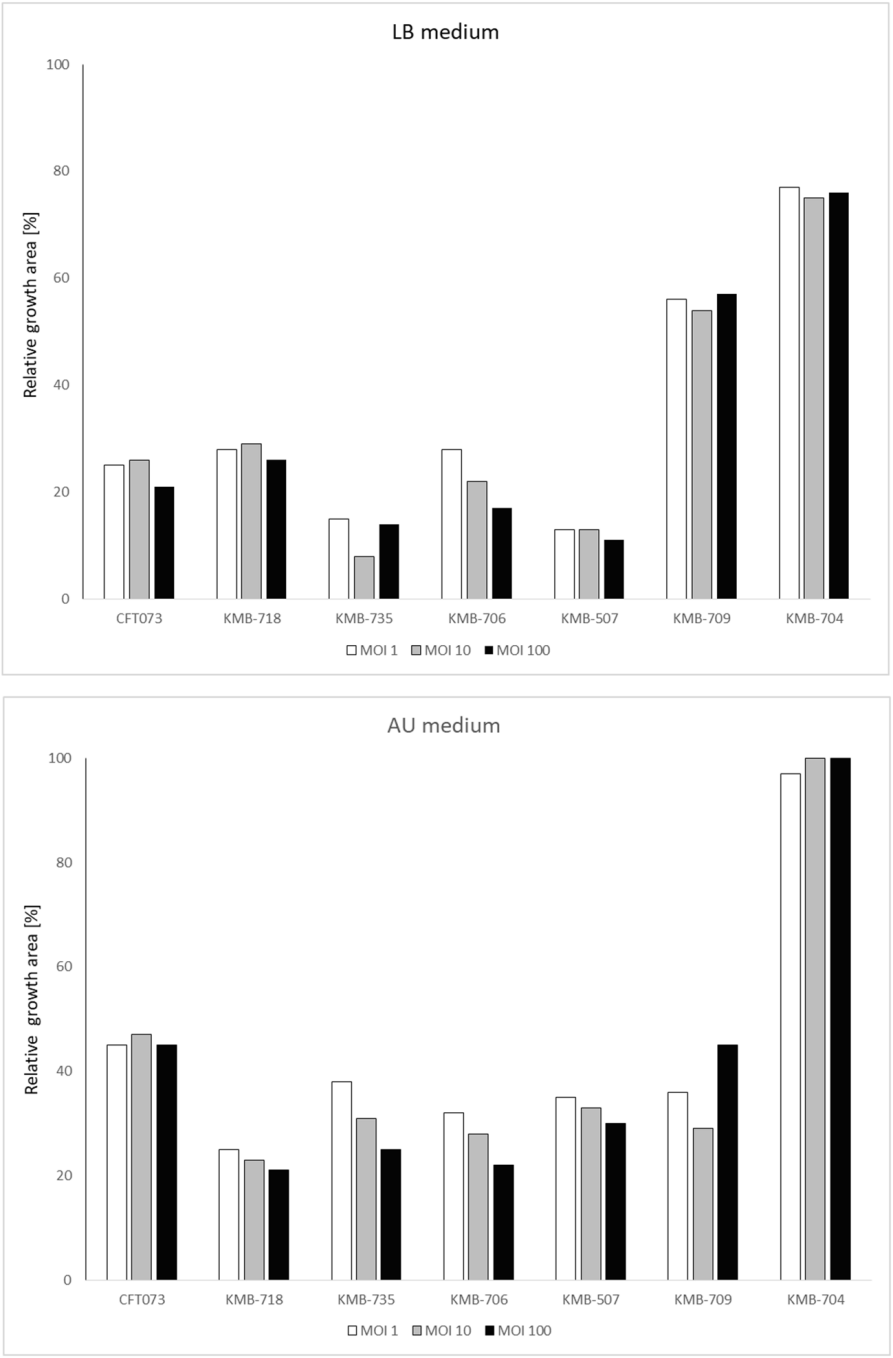
Overall bacterial growth inhibition in the presence of phage cocktail in LB and AU media. Growth inhibition was observed as an area of optical density in cocktail samples divided by an area of optical density in control samples without phages. Three different MOIs were used: 1 (white), 10 (grey), and 100 (black)

Inhibition of *E. coli* growth was measured in microtiter plates for 24 hours using two media: rich LB and artificial urine medium (AU). We observed great variations in the degree of bacterial growth inhibition by the phage cocktail. In six susceptible strains, almost complete suppression of bacterial growth was observed in the first 5–22 hours of incubation. After this period, the optical density started to increase but did not reach the level of the positive control (Fig. 3). No significant difference between cocktails with an EOP of 1, 10, and 100 was observed. Overgrowth of the culture did not correlate with the number of phages to which the strain was susceptible. Instead, it appeared to be a random process, and for some strains (especially *E. coli* KMB-735), great variations between parallel growth experiments were observed. Some differences in cocktail efficiency between nutrient-rich LB medium and artificial urine medium were also observed. Overall, the level of inhibition of six susceptible strains by the cocktail at 24 hours postinfection reached 43–92% in LB medium and 53–79% in AU medium (Fig. 4). In AU medium, similar levels of inhibition were observed for strains that were pre-adapted by overnight cultivation in AU medium as for LB-grown inoculum (data not shown). In the case of the phage-resistant strain *E. coli* KMB-704 (ST69), the phage cocktail did not affect its growth, although the optical density decreased slightly during late phase of cultivation in LB medium, with an overall reduction of 76%.

## Discussion

Phages for phage therapy applications can be easily isolated from the environment [12, 33, 34]. However, not every phage is suitable for therapy. Only phages with an appropriate host range should be used, and this may vary depending on the country or environment, as pathogenic strains are adapted to local conditions. It is also necessary to use strictly lytic phages that do not have genes encoding virulence factors or conferring antibiotic resistance. Such well-characterized phages are deposited in phage banks, which serve as a source of phages for therapeutic or industrial applications [16, 34].

In the present study, we characterized 16 coliphages isolated from wastewater as candidates for UPEC control agents. Two of the phages, vKMB22 and vKMB26, which were isolated and partially characterized in our previous study [17], were also included. The phages showed a variable range of host specificity on a panel of UPEC strains (Fig. 1). The broadest host specificity was observed for phages belonging to the family *Straboviridae*. The *Autographiviridae* and *Stephanstirmvirinae* members were able to infect a moderate number of hosts, and other phages possessed narrow specificity. This observation is consistent with the fact that *Straboviridae* (T-even) phages are among the most frequently isolated *E. coli* phages and are used in various applications [35–38]. However, we also observed up to twofold differences in the host range between closely related *Tequatrovirus* phages. Phage sensitivity is influenced by many factors. For example, a point mutation in the phage adhesin has been reported to cause a substantial decrease in the host spectrum of *Straboviridae* phage Pet-CM3-4 infecting members of the genera *Cronobacter* and *Enterobacter* [39].

We observed that the *E. coli* strains that were susceptible to the phages in this study belonged predominantly to the phylogenetic groups B2 and D. Limited phage sensitivity was observed with strains of phylogroups A and B1 (Fig. 1A). Such preferences for certain *E. coli* phylogenetic groups were observed previously in phages isolated from poultry meat or feces, which predominantly infected members of phylogroup A, B1, or D [40]. Similarly, phages isolated from wastewater in Germany showed increased virulence against phylogroup B2 [38]. It is not obvious why we were unable to isolate phages with a broad host range within phylogroups A or B1 despite the fact that six of the phages were initially propagated on indicator strains of these phylogroups and some of them showed moderate activity against B2 strains. In general, the habitat of a bacteriophage is dependent on its host bacteria, and our results might reflect the predominance of B2 strains among *E. coli* in wastewater [5, 7].

Genome analysis of the phages revealed that they all belonged to known genera, but most of them did not belong to established species, as they differed at > 95% of their nucleotide positions from phages with sequences in the GenBank database [32]. Phages vKMB26 (genus *Tequatrovirus*) and vKMB47 (genus *Vectrevirus*) were the exceptions, as they were almost identical to phages isolated recently from wastewater in Germany [38]. Those phages, like the ones in this study, exhibited a broad host range on UPEC strains. Isolation of phages of the same species in different countries implies that the global spread of UPEC strains, such as sequence type ST131 [5, 21], has favored the distribution of phages adapted to them.

A comparison of the genome sequences of the *Straboviridae* phages revealed mosaicism, which is a typical feature of the T-even group (Fig. 2) [41]. Many of these variable genes are responsible for overcoming host defenses, such as the dCMP hydroxymethylases, glucosyl transferases, and orphan DAM methylases, which are responsible for extensive phage DNA modifications, and internal head proteins (IP) conferring protection against GmrSD endonuclease [42, 43]. The genome of phage vKMB26 encodes an extra beta-glucosyltransferase gene with no homology to previously characterized genes, which could provide the phage with an unusual DNA glucoslylation pattern. This modification, together with a unique sequence of distal tail fibers, may explain the extended host range of vKMB26.

Phage vKMB38 (genus *Mosigvirus*) similarly to reference phage RB69, does not encode a glucosyltransferase, suggesting the presence of hmC without glucosylation. However, a putative glycosyltransferase responsible for arabinosyl-hmC modification in phage RB69 [44] was also found in vKMB38. Despite some properties that are typical of highly virulent phages (e.g., the presence of a *dmd* antitoxin gene), vKMB38 could infect only 17% of the UPEC strains tested and is therefore the member of the family *Straboviridae* with narrowest host range in this study.

Two phages of the genus *Vectrevirus*, family *Autographiviridae*, vKMB14 and vKMB47, have a typical T7-like organization and differ mainly in the tail spike region, and this might account for differences in their host spectrum. In both genomes, we identified two tail spike genes encoding polysaccharide depolymerases with different specificity. Such an arrangement is frequently found in *Autographiviridae* phages [45]. The first spike in both genomes has a high degree of similarity to the K5 lyase of *Escherichia* phage K1-5. The second tail spike in vKMB14 shows similarity to the pectate lyase domain [46], but with low amino acid sequence similarity to other phage tail spikes (< 43%), giving it unique specificity. The other phage, vKMB47, was found to be highly similar to the recently described *Escherichia* phage vB_EcoP-101101UKE1 [38, 46], and a high level of similarity was also found in the second tail spike gene.

The remaining six phages were classified into five genera, possessed lower host specificity, and belonged to taxa that are more rarely represented in DNA databases. Of these, phage vKMB37 (genus *Justusliebigvirus*) was able to infect the largest number of strains. This phage is closely related to phage phi92 [47], but it contains a different RBP gene. As vKMB37 infects strains with different types of capsular polysaccharide, it seems that it is not as capsule-dependent as phi92. Like phi92, vKMB37 carries alx-like *ter* cluster genes, which are involved in bacterial stress response systems [47, 48]. The function of these genes during the phage life cycle is unknown.

The two phage isolates belonging to the genus *Kagunavirus*, vKMB35 and vKMB36, were found to be distantly related to each other (Fig. 2). Both of these produced plaques with turbid halos and encoded polysaccharide depolymerases that did not show amino acid sequence similarity to the sialidase of K1G or to any other depolymerases. It is interesting that vKMB36 seems to contain a 900-bp intein in the minor head protein gene. Inteins are mostly found in DNA-binding proteins, such as terminase [49], and they were also detected recently in the capsid proteins of *Salmonella* phages [49, 50]. Surprisingly, there are no reports of inteins in *E. coli* or in coliphages, suggesting that vKMB36 obtained the intein from another host, probably from *Salmonella*.

Overall, we can conclude that isolated phages, except for vKMB25, possess genome properties suitable for therapeutic use, lacking known virulence or antibiotic resistance genes.

Based on our previous results, the six phages with the best properties were combined into a phage cocktail, the antibacterial activity of which was measured *in vitro*. Phage cocktails represent the best way to prepare phage preparations with sufficient host specificity [40, 51]. In most cases, the selection of phages for a cocktail is based on their host specificity and growth parameters, based on empirical rules [52]. Four *Straboviridae* and two *Autographiviridae* phages were combined for this study. These phages belong to the species found most frequently in the environment and whose members have been used in previous phage therapy studies [51, 53]. The two *Autographiviridae* phages are predicted to produce polysaccharide depolymerases, which could potentially decompose bacterial capsules or biofilms, and thus could improve the efficiency of the cocktail. The phages selected to the cocktail possessed broad host specificity on double agar and excellent growth properties when measured on the original propagating strain (Table 2).

When the activity of the cocktail was tested in liquid medium we did not observe bacterial growth during the first hours of cultivation, but partial regrowth was observed at later times (Fig. 3). Development of phage resistance did not correlate with the number of phages infecting a particular strain on double agar but instead appears to have been a random process, and for some strains, great variations between parallel experiments were also observed. Interestingly, the highest growth suppression was observed for two strains, KMB-706 and KMB-507, which were sensitive only to phages of the family *Straboviridae*. In the present study, bacterial receptors were not identified, and it is therefore possible that multiple phages in the cocktail bind to the same receptor, making co-selection of resistance to these phages possible. Our observations are consistent with a previous report that the spot method tends to indicate greater phage sensitivity than the microtiter assay, which is better for monitoring host-phage interactions [31].

The cocktail prepared in this study was efficient in LB as well as in moderately acidic artificial urine medium, which mimics conditions present in UTIs [29, 30]. It is interesting that, in some cases, the efficiency of the cocktail in artificial urine was even better than in LB. These results indicate that our phage cocktail has the potential to inhibit bacteria during infection, and these phage isolates will therefore be preserved in the national phage bank, serving as valuable resources for therapeutic applications.

## Data Availability

DNA sequences were deposited in GenBank, the other data are presented in the manuscript.
